# Regulation of Subunit-Specific Germinal Center B Cell Responses to the HIV-1 Envelope Glycoproteins by Antibody-Mediated Feedback

**DOI:** 10.3389/fimmu.2017.00738

**Published:** 2017-06-30

**Authors:** Mattias N. E. Forsell, Linda Kvastad, Saikiran K. Sedimbi, John Andersson, Mikael C. I. Karlsson

**Affiliations:** ^1^Division of Immunology, Department of Clinical Microbiology, Umeå University, Umeå, Sweden; ^2^Department of Microbiology, Tumor and Cell Biology, Karolinska Institutet, Stockholm, Sweden; ^3^Department of Medicine, Solna, Karolinska Institutet, Stockholm, Sweden

**Keywords:** epitope-specific antibodies, regulation of germinal centers, HIV-1, envelope glycoproteins, epitope-specific B cells

## Abstract

The regulation of germinal center (GC) B cell responses to single epitopes is well investigated. How monoclonal B cells are regulated within the polyclonal B cell response to protein antigens is less so. Here, we investigate the primary GC B cell response after injection of mice with HIV-1 envelope glycoproteins. We demonstrate that single GCs are seeded by a diverse number of B cell clones shortly after a single immunization and that the presence of Env-specific antibodies can inhibit the development of early GC B cells. Importantly, the suppression was dependent on the GC B cells and the infused antibodies to target the same subunit of the injected HIV-1 envelope glycoproteins. An affinity-dependent antibody feedback has previously been shown to regulate GC B cell development. Here, we propose that this antibody-based feedback acts on GC B cells only if they target the same or overlapping epitopes. This study provides important basic information of GC B cell regulation, and for future vaccine designs with aim to elicit neutralizing antibodies against HIV-1.

## Introduction

There is abundant evidence that some HIV-1-infected patients develop broadly neutralizing antibodies (bNabs) at the chronic stage of the infection (1, 2). This demonstrates that the human immune system is, under certain circumstances, capable to produce antibodies that may be useful if they could be re-elicited by vaccination. Being the only virally derived component on the outside of the virion, it is not surprising that known bNabs target the HIV-1 envelope glycoproteins (Env) (3). It has been postulated that humoral immune responses to immunodominant regions of Env may suppress responses to less immunogenic regions, and that this could explain why bNabs are infrequently elicited during infection and has, to date, not been elicited by vaccination. Clearly, a better understanding of the regulatory processes for epitope-specific regulation and maturation of B cell responses is of great importance for the development of improved vaccine strategies.

Immunization with recombinant proteins in adjuvant generates T-dependent humoral immune responses that are characterized by the formation of germinal centers (GCs). In GCs, antigen-specific B cells undergo affinity maturation and differentiation into memory B cells and Ab-secreting plasma cells [reviewed in Ref. (4)]. The resulting polyclonal Ab response comprises a number of different antibodies that each target a distinct epitope surface on the injected protein antigen (5). In the GC, B cell clones that target the same epitope on model antigens are competitively regulated and there is a bias for survival of high-affinity clones (6–8). It was demonstrated that B cell clones with a high-affinity BCR are better at presenting antigenic peptides to Tfh than are B cells with low affinity, and therefore gain a competitive advantage (9), and the importance of robust Tfh responses for the generation of neutralizing antibodies against HIV-1 has been extensively discussed elsewhere (10). However, even within single GCs a wide range of intra- and inter-clonal affinity maturation of B cells occur (11, 12). It is therefore possible that regulatory mechanisms exist to allow for clonal expansion and maturation of B cells with different epitope specificity after challenge with physiologically relevant multi-epitope proteins, such as HIV-1 Env. By dampening the ability of B cells to recognize the immunodominant V3-region on Env, we have previously shown that antibody and plasma cell responses to distinctly different epitope regions were independently regulated after repeated immunizations with recombinant soluble HIV-1 Env in mice (13). Similar results were subsequently found when instead immunosilencing the trimerization domain of Env (14). These findings were not unique to Env, as similar observations had previously been described for a number of therapeutic proteins, including *Pseudomonas* exotoxin A [reviewed in Ref. (15)]. Immunodominance may therefore be driven by a mechanism that is largely independent of inter-clonal competition and additional regulatory mechanisms might play a significant role for the regulation of B cell clones with distinct BCR specificities within the polyclonal response after immunization.

For decades, it has been known that IgG can feedback regulate the humoral immune response, and that this is dependent on the nature of the antigen and subclass [reviewed in Ref. (16)]. It was demonstrated that IgM could mediate inhibition of GC B cell responses by direct binding to antigen, thereby occluding it from recognition by antigen-specific BCRs on B cells (17). Since IgM is readily elicited early during the development of T cell-dependent GC B cell responses, it is unlikely to provide a strong inhibitory effect on GC B cells under physiological conditions. However, an antibody-mediated feedback mechanism that is dependent on the binding specificity of IgG could potentially explain our results where independent expansion of epitope-specific plasma cell responses to HIV-1 Env was observed (13).

A single injection with Env in adjuvant was not sufficient to induce potent Env-specific IgG-secreting plasma cells in mice, rabbits, and non-human primates (13, 18, 19). If antigen-specific GC B cells had been developed at the same time point, this would allow us to investigate how Env-specific GC B cell responses develop without the interference of endogenously produced antigen-specific antibodies. According to this rationale, we set out to define the characteristics of the GC B cell response after one injection of Balb/C mice with Env, and then to address if an antibody-mediated feedback had potential to regulate GC B cell responses in an epitope-specific manner.

## Materials and Methods

### Recombinant Proteins

The design and cloning of trimeric soluble recombinant envelope glycoproteins Env and monomeric gp120 for injection, and trimeric Env, gp120, and gp120ΔV3 for site-specific biotinylation has been previously described (20, 21). All recombinant proteins were produced by using the FreeStyle™ 293 Expression system (Invitrogen) and purified by sequential lectin and his-tag affinity chromatograph (22). Site-specific biotinylation was performed by treating AviTagged recombinant Env and gp120 with biotin-protein ligase (GeneCopoeia, Rockville, MD, USA) (20).

### Immunizations

For injections, 10 μg of Env or gp120 was emulsified in Imject™ Alum adjuvant (Thermo Fischer Scientific) and 7- to 10-week-old BALB/c mice were injected *via* the intraperitoneal route. To generate immune serum to Env or gp120, groups of six mice were injected with recombinant Env or gp120 in Imject™ Alum adjuvant two times at a 2-week interval, and serum was collected 2 weeks after the last injection. Serum from mice injected with Adjuvant alone was used as control. Mice were kept at the animal facility at Department of Microbiology, Tumor and Cell Biology, Karolinska Institutet or at the Umeå Center for Comparative Biology, Umeå University, Sweden.

### Immunohistochemistry and Laser Microdissection

For immunohistochemistry and laser capture microdissection of GC structures, 8 μm sections of OCT embedded spleens were fixed on super frost plus glass slides (Thermo Scientific) or on PPS membrane slides (MicroDissect GmbH), and fixed using ice-cold acetone. For subsequent laser microdissection, we chose the mid section of a three consecutive 8 μm sections that all demonstrated a GC structure of same shape and relative location in the spleen. To inhibit non-specific binding, sections were treated with 5% goat serum (Dako) and subsequently treated with Avidin/Biotin blocking kit. Slides were then stained with FITC-conjugated anti-IgD (BD Pharmingen) and biotinylated peanut agglutinin (PNA) followed by Alexa555-conjugated streptavidin (Thermo Fisher Scientific). Confocal microscopy was performed on the glass slides with a DM IRBE system (Leica). Laser microdissection was performed on PPS membrane slides in a LMD7000 system (Leica). Single GC structures were defined as PNA^+^, IgD^−^ areas inside splenic follicles (IgD^+^, PNA^−^) in the center section of each spleen, and collected in RLT buffer for subsequent mRNA extraction.

### Flow Cytometry and Cell Sorting

Single-cell suspension of splenocytes was achieved by passing spleen through a 70-µm nylon mesh. RBCs were subsequently lysed with hypotonic ammonium chloride solution for 1 min, and the remaining cells were washed and resuspended in complete RPMI 1640 medium (Sigma) containing 5% FBS, 50 µM 2-ME, 2 mM l-glutamine, 100 U/ml penicillin, and 100 µM streptomycin. Where applicable, splenocytes were enumerated by flow cytometry using AccuCheck Counting Beads (Life Technologies). The amount of live cells in samples was determined by using a Live/Dead aqua viability kit (Thermo Fischer Scientific). Antibodies used for stainings were FITC-conjugated anti-GL7 antigen, PerCP.Cy5.5-conjugated anti-IgD, PE-conjugated anti-CD95, and Pacific Blue-conjugated anti-B220 (all BioLegend). To determine antigen specificity, cells were incubated with 10 μg/ml biotinylated Env trimers, gp120 trimers, or gp120ΔV3 trimers and subsequently with APC-conjugated streptavidin. Data were collected on a BD LSRFortessa™ X20, and cell sorting was performed on a BD Facs Jazz™ (All BD Biosciences). Analysis of flow cytometric data was performed using FlowJo (FlowJo, LLC).

### B Cell Receptor Fragment Analysis

The B-cell repertoire was assessed by spectratyping of VDJ regions of heavy chain families 1, 2, 3, 5, 6, and 7. Briefly, mRNA from tissues was extracted with an RNAeasy kit (Invitrogen), and corresponding cDNA was then generated using iScript (BioRad), according to the manufacturer’s instructions. Previously published primers for amplification of the VDJ region (with focus on the uniqueness of the CDR3) of the variable region heavy chain (Vh) families 1 and 2 of mice were used to amplify the target regions [Vh1 forward: TCCAGCACAGCCTACATGCAGCTC; Vh2 forward: CAGGTGCAGCTGAAGGAGTCAGG; and Jrev (common primer in the JH-region): CTTACCTGAGGAGACGGTGA] (23, 24). The amplifications were performed in a total volume of 20 µL, using 2× GoTaq (Promega), 2 µL (1 µM final) of each primer, and 2 µL of cDNA. After 1 min at 95°C, amplification was performed for 40 cycles as follows: 30 s at 95°C, 30 s at 55°C, and 1 min 30 s at 72°C, and ended with a step of 10 min at 72°C. To label the amplified fragments, 5 µL of each P CR product was mixed with 0.5 µM 6-fluorescein amidite (FAM)-labeled Jrev-primer and 5 µL GoTaq and subjected to 10 runoff cycles as follows: 2 min at 95°C, 2 min at 55°C, and 20 min at 72°C, and ended with a 10-min step at 72°C. FAM-labeled products were then processed on an ABI3130 Genetic analyzer (Applied Biosystems). Data were analyzed using PeakScanner v1.0 software (Applied Biosystems). Each peak in the resulting histogram represents one or many B cell clones with identical nucleotide length of the VDJ region of a certain Vh family. For an approximation of a distinct number of clones present in a single GC, a stringent cutoff of 1,000 response units (RUs) was applied to select for dominant clones. The relative dominance of the single fragment with the highest RU value in a GC was calculated with respect to the sum of RUs of all detected fragments in the same (%dominance = RU_dominant fragment_ × 100/Σ RU_all fragments_).

### Enzyme-Linked Immunosorbent Assay

High-protein-binding MaxiSorp plates (Nunc) were coated with 100 or 200 ng/well of recombinant Env or gp120 at 4°C overnight. The coated plates were blocked with 2% fat-free milk in PBS. After washing (PBS, 0.05% Tween-20), serum was added at different concentrations. The wells were then incubated with HRP-conjugated anti-mouse IgG or IgM (Southern Biotech). After washing, a colorimetric HPA substrate containing 3,3′,5,5′-tetramethylbenzidine (Invitrogen) was added. Adding one volume of 1 M H_2_SO_4_ stopped the enzymatic reaction, and OD was read at 450 or 450–620 nm. All incubations were performed at room temperature for 1 h, unless otherwise stated.

### Statistical Analysis

Statistical analysis was performed using GraphPad Prism V5.04 (GraphPad Software). Data sets were first analyzed with the D’Agostino and Pearson omnibus normality test. Sets conforming to normal distribution were then analyzed further using ANOVA or non-paired two-tailed Student’s *t*-test to determine the significance of observed differences. Data sets not exhibiting normal distribution were analyzed using a non-parametric ANOVA, Mann–Whitney *U* test, or the Wilcoxon matched-pairs signed-rank test.

### Ethics Statement

All animal experiments were pre-approved and performed in accordance with the Swedish Animal Welfare Act under protocols Dnr 234/12-dnr 11/13 (approved by Stockholms Norra djurförsöksetiska nämnd, Sweden) and Dnr A 59-15 (approved by Umeå försöksdjursetiska nämnd, Sweden).

## Results

### GC B Cell Responses after Immunization with HIV-1 Env

**Figure 1:**
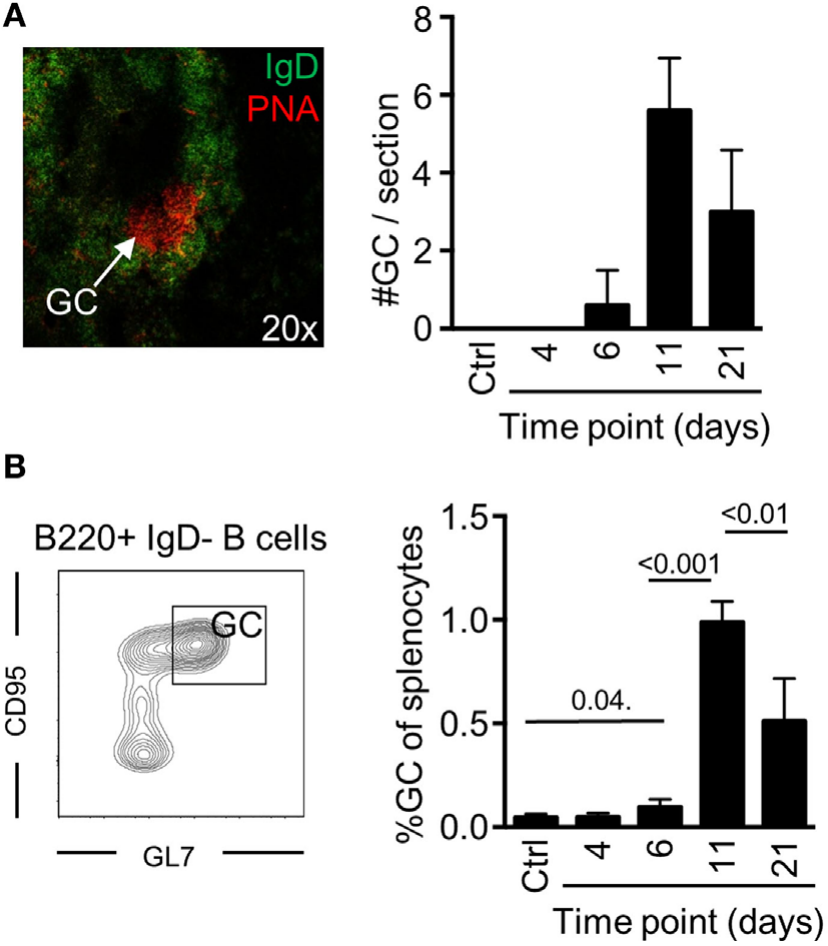
Germinal center (GC) B cell development after a single injection of mice with Env. **(A)** The presence of GC structures was assessed by immunofluorescence microscopy as distinct PNA^+^/IgD^−^ areas (red) within follicles (PNA^−^/IgD^+^, green) in spleen sections of mice (representative image, left panel). The number of GCs in spleen sections from individual mice was enumerated at the indicated time points (right panel). **(B)** Flow cytometric quantification for the frequency of GC B cells (B220^+^IgD^−^GL7^+^CD95^+^) of total splenocytes is shown at the indicated time points. *N*_(adjuvant, d4)_ = 4 animals; *N*_(d6, d11, d21)_ = 5 animals.

To determine if potent GC B cell responses occur after a single injection with Env, we devised an injection regimen to characterize the development of GC B cells after immunization with Env in Imject Alum™ adjuvant. By immunofluorescence microscopy, we found that distinct GC formation (PNA^+^IgD^−^) could be detected on day 6 by histology (Figure 1A). The numbers of splenic GCs had significantly increased on day 11, but were reduced in numbers again by day 21 after the immunization. To quantify our findings, we assessed the frequency of splenic GC B cells (B220^+^IgD^−^CD95^+^GL7^+^) by flow cytometry at the same time points. Consistent with our histological results, we found that the overall frequency of GC B cells had reached detectable levels at day 6, that a major expansion had occurred between days 7 and 11 (Figure 1B). We could also quantify the overall reduction of GC B cells between days 11 and 21 after immunization Collectively, these data verify that GC B cell responses develop after a single injection of mice with Env in adjuvant.

### Clonal Expansion and Contraction of B Cells in GCs after Immunization with HIV-1 Env in Mice

**Figure 2:**
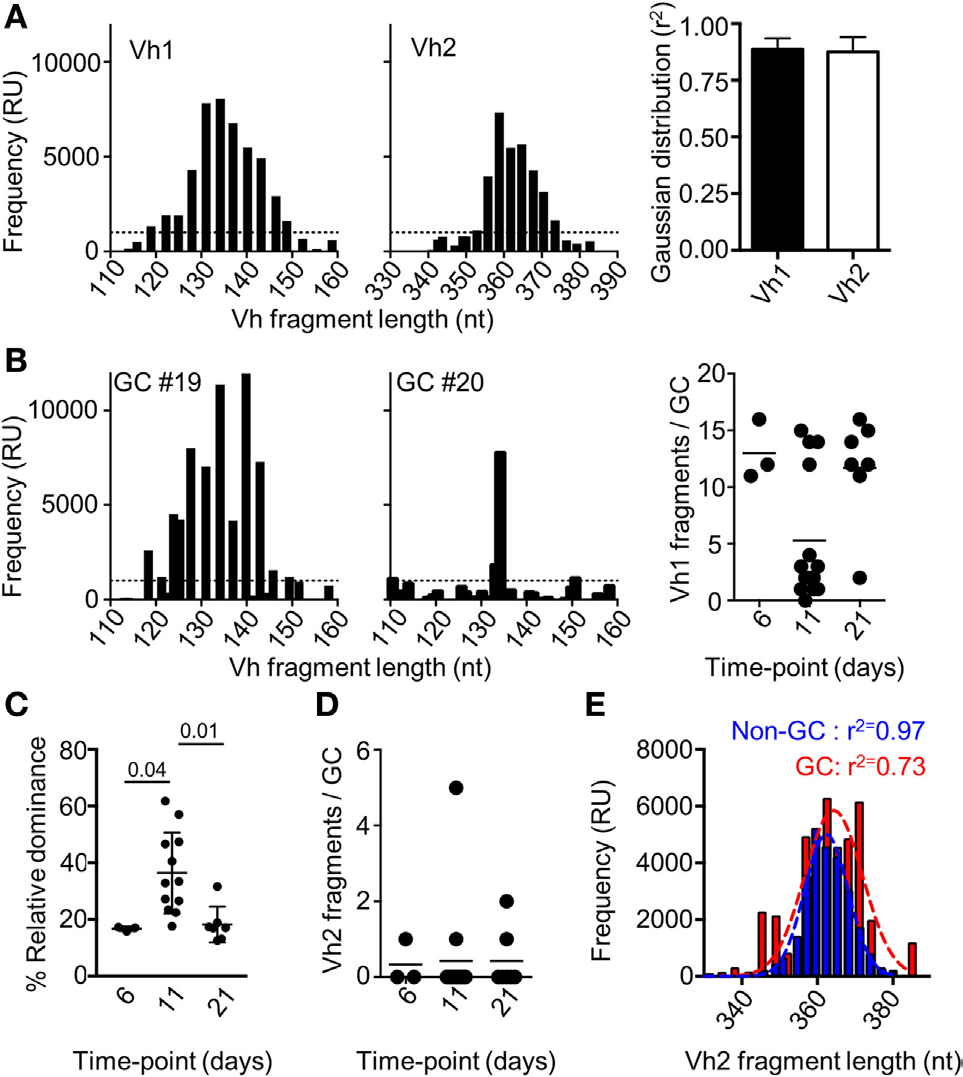
Fragment analysis for the presence of B cell clones of the Vh1 or Vh2 families. **(A)** Splenocytes from uninjected mice (*N* = 3) were assessed for the number of Vh1 (left panel) or Vh2 (mid panel) fragments of different nucleotide length that could be amplified. The distribution of the fragments with respect to their frequency was assessed for adherence to a Gaussian distribution (right panel). **(B)** Fragment analysis of the Vh1 family in single germinal centers (GCs) after isolation by laser capture microdissection. Shown are representative histograms of the Vh1 distribution in polyclonal GCs (left panel) and relatively monoclonal GCs (mid panel), 11 days after injection with Env. Enumeration of how many distinct Vh1 fragments that could be found in each of the isolated single GCs on day 6 (*N* = 3), day 11 (*N* = 14), and day 21 (*N* = 7) was performed (right panel). **(C)** The relative dominance of the VDJ fragment with the highest frequency among all detected Vh1-family VDJ fragments is shown. **(D)** Shown is an enumeration of how many distinct Vh2 fragments that could be found in each of the isolated single GCs at different time-points. **(E)** GC B cells (B220^+^IgD^−^GL7^+^CD95^+^, red) and non-GC B (B220^+^IgD^−^GL7^−^CD95^−^, blue) cells were sorted and assessed for the frequency and number of amplified Vh2-family VDJ fragments. The *r*^2^-value indicates the adherence to a Gaussian distribution of different fragments with respect to their relative frequency.

It has been previously shown that the number of dominant B cell clones in a single GC could vary after immunization with other recombinant proteins (11, 25–27). If a monoclonal B cell population seeds separate GCs after immunization with Env, this could explain the lack of competition between the responses to different epitopes within the antigen. To address this, we isolated single GCs (IgD^−^PNA^+^) by laser capture microdissection and approximated the relative clonality of these at different time points after immunization. This was done by assessing the number of VDJ regions of heavy chain (Vh) with variable nucleotide lengths that could be amplified from spleen sections from mice shown in Figure 1. We focused on the Vh1 family that cover a large part of the total Vh-repertoire of mice, and on the Vh2-family that cover a limited part of the Vh-repertoire (28). To validate the method, we first amplified Vh1 and Vh2 VDJ regions from a sectioned spleen. As expected, we could amplify a large number of Vh1 and Vh2 VDJ fragments from the polyclonal population of B cells in the spleen section, and the frequency of amplified fragments were closely adhering to a Gaussian distribution with respect to fragment length (Figure 2A). Focusing on the Vh1 family, we could amplify a large number of VDJ fragments from single GCs at days 6 and 21 after injection with Env, whereas a significant number of GCs contained a relatively few Vh-1 fragments at day 11 (Figure 2B). This suggested that significant clonal selection had occurred between days 6 and 11, but that the GC B cell population had then diversified with respect to fragment lengths between days 11 and 21 after the immunization. To quantify this, we investigated the relative dominance of the most abundant Vh1 VDJ fragment among all amplified Vh1 VDJ fragments from single GCs over time. Consistent with polyclonal GC B cell populations, the dominance of a single VDJ fragment in separate GCs was on average 16.5% (range: 16–17%) or 18% (range: 12–31%) of all VDJ fragments on days 6 or 21 after injection (Figure 2C). By contrast, the average dominance of a single VDJ was 36% (range: 18–61%) on day 11 after the injection. This supports that GCs at peak response have reduced B cell clonality, but that fully monoclonal GCs were rare. Instead, GCs at peak response display variable degrees of clonal dominance. A similar variation of clonal dominance in single GCs was previously shown after injection of mice with chicken gamma globulin, *Bacillus anthracis* protective antigen and influenza hemagglutinin (11, 12).

Detection of clones from the Vh2-family in single GCs was rare at all time-points and when detected, comprised up to five fragments (Figure 2D). By contrast, a large number of clones of the Vh2-family could be detected after flow cytometric sorting of GC B cells 11 days after immunization of mice with Env (Figure 2E). The conflicting data are likely explained by the presence of a cross-section of all responding GC B cell clones after the flow cytometric sorting, whereas the laser capture microdissection allowed for analysis of GC B cell clones from single GCs. A Gaussian distribution analysis of the sorted cells revealed that non-GC Vh2 B cell clones were normally distributed with respect to their BCR length (*r*^2^ = 0.97). By contrast, the distribution of Vh2-related GC B cells was slightly skewed (*r*^2^ = 0.78). Even though seeding and recruitment of Vh2-family B cells to single GCs was low in comparison to Vh1 clones, biased selection of GC B cell clones had occurred, if assessed on a global level.

### Development of Antigen and Epitope-Specific GC B Cell Responses after Immunization of Mice with HIV-1 Envelope Glycoproteins

**Figure 3:**
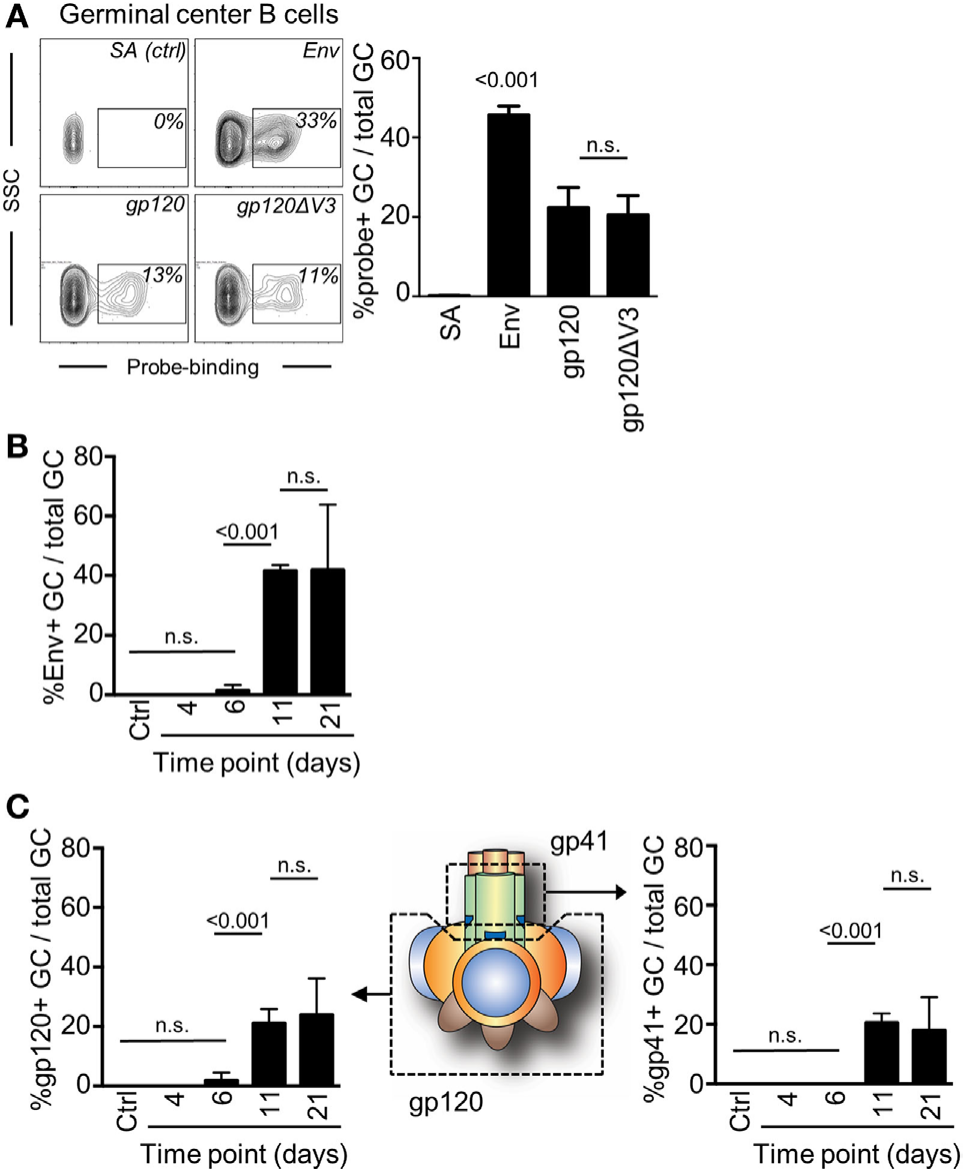
Detection of antigen and subunit-specific germinal center (GC) B cell responses after a single injection of mice with Env. **(A)** The frequency of GC B cells (B220^+^IgD^−^GL7^+^CD95^+^) 11 days after injection which were able to bind biotinylated Env, gp120, or gp120ΔV3 was assessed by flow cytometry after addition of APC-conjugated streptavidin. Staining of cells with APC-conjugated streptavidin (SA) in the absence of Env was used verify the specificity of the binding. **(B)** The frequency of Env-specific GC B cells of total GC B on days 6, 11, and 21 after injection of mice with Env is shown. **(C)** The frequency of GC B cells (B220^+^IgD^−^GL7^+^CD95^+^) that could bind to the gp120 subunit of Env was assessed in a similar manner (left panel). By subtraction of the gp120-specific GC B cells from the total Env-specific GC B cells, we could also determine the proportion of gp41-specific GC B cells that had been induced at the same time points (right panel). *N* = 4–5 animals per group.

A requirement for an investigation to understand if antibodies can mediate a feedback to regulate epitope-specific GC B cells was that we could also measure GC B cell responses to two distinctly different regions of Env. Here, we took advantage of a probe-based system that we had previously used to enumerate subunit-specific plasma cell responses after repeated immunizations with Env (20). To test this system, we first assessed the capacity of splenic GC B cells to bind to Env, the gp120 subunit, or to a gp120 subunit that lack the variable region 3 (gp120ΔV3) 11 days after injection with Env. We found that an average of 45% of GC B cells was specific for Env, and that approximately 50% of those could bind to both gp120 and the gp120ΔV3 probes (Figure 3A). Importantly, we had previously shown that repeated injection of Env into mice did produce significant B cell responses to the non-exposed inside of Env trimers (20). Therefore, the Env-specific response was evenly distributed between epitopes that span the gp120 or the gp41 subunits, whereas no significant response had developed against the variable region 3 at this time point.

Next, we assessed changes in frequency of Env-binding GC B cells over time by flow cytometry. We injected mice with Env and found that GC B cells had reached sufficient numbers and affinity for Env to be detectable in our analysis after 11 days (Figure 3B). This suggested that significant proliferation, antigen-specific affinity maturation, and selection of GC B cells had occurred during the second week after the injection. The frequency of B cells that could bind to the Env-based probe was not significantly changed between days 11 (median: 41%) and 21 (median: 50%).

Subtracting gp120-specific responses for the total Env-specific response allowed us to determine the specific response to the gp41 subunit of Env. As expected, gp120 and gp41 subunit-specific responses developed with the same kinetics as the total Env-specific response and required between 7 and 11 days to develop sufficient affinity for detection (Figure 3C). No further increase in the frequency of gp120- or gp41-binding GC B cells had occurred between days 11 and 21 after a single immunization of mice with Env. Collectively, gp120-specific GC B cells accounted for a median of 51% (day 11) and 58% (day 21) of total Env-specific GC B cells. Consistently, gp41-specific GC B cells accounted for the remaining 49% (day 11) and 42% (day 21) of total Env-specific GC B cells.

### Regulation of Subunit-Specific GC B Cell Responses to the HIV-1 Envelope Glycoproteins

**Figure 4:**
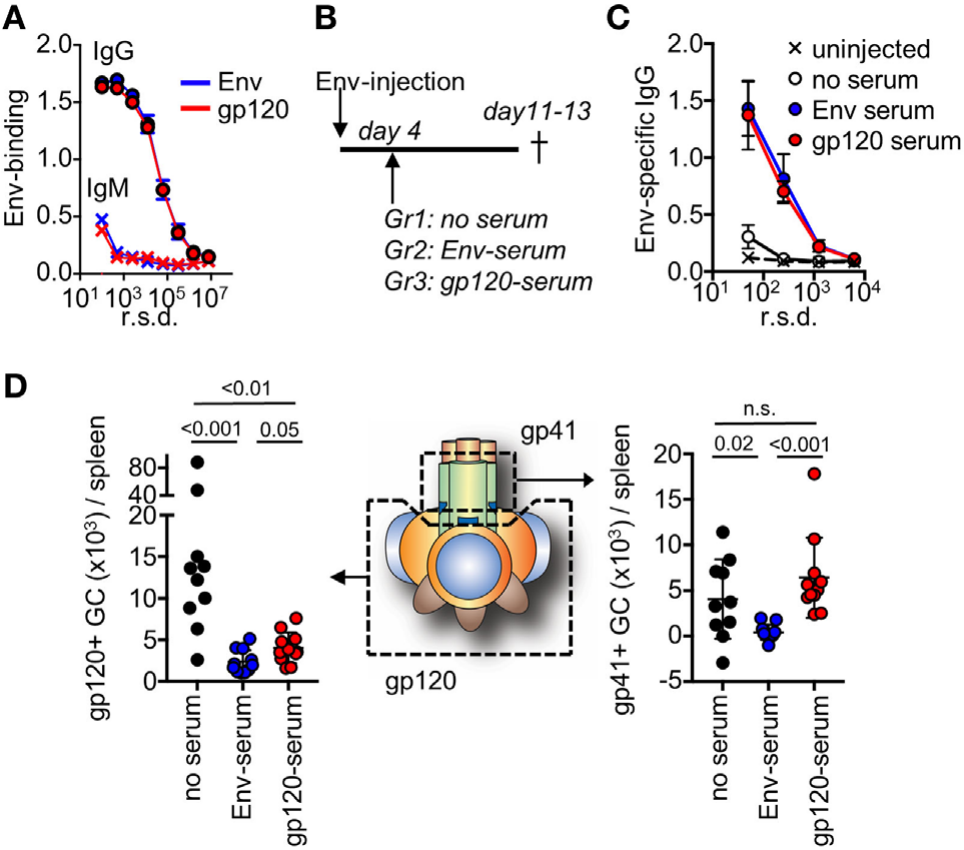
After repeated injections with either Env or the subunit gp120, serum was harvested from mice and assessed for binding to Env. **(A)** Shown is IgG or IgM in serum pre-normalized for Env binding, as assessed by ELISA. **(B)** Mice (*N* = 5–6/group) were injected with Env and anti-Env or anti-gp120 serum was infused at the indicated time point. The mice were terminated on days 11–13 after start of the experiment. Control mice (*N* = 4–5/group) did not receive a serum infusion. **(C)** Assessment of Env-specific IgG by ELISA in serum from respective groups 13 days after injection with Env. **(D)** Detection of gp120 and gp41-specific germinal center (GC) B cells in groups of mice that had received Env-specific or gp120-specific serum, or no serum on day 4 after the Env injection. Shown is the accumulated data from two separate experiments.

To study feedback regulation, we generated serum by repeated injections of mice with soluble Env or with gp120, that either contained antibodies to both the gp120 and gp41 subunits (Env injection) or only to the gp120 subunit (gp120 injection). We subsequently normalized the harvested serum so that both had a similar binding capacity to Env with regard to IgG and IgM (Figure 4A). Respective serum was then further diluted 2× in PBS and 200 μl was infused into mice that had been immunized with Env 4 days earlier (Figure 4B). We chose this time point to allow for similar initiation of the GC response toward Env in all groups prior to the serum infusion (29), and that it was just before GC B cells could be detected by flow cytometry (Figure 1A). Moreover, it would allow for similar trafficking and retention of Env to the network of follicular dendritic cells in GC for the first 4 days after immunization (30, 31). Since the injected serum levels was below those that can be induced by repeated Env injections into BALB/c mice, the potential regulatory function of antibodies on GC B cell responses likely mimic that of the endogenous high-affinity anti-Env antibody response after it has been generated.

Two weeks after immunization of mice with Env, we could detect low-levels of circulating Env-specific IgG after one injection (Figure 4C). This represented the background levels of the endogenous response at a time point when Env-specific GC B cells had developed. In both the serum-infused groups, we found distinct levels of circulating Env-binding IgG. Since these levels were significantly higher than that of the endogenous response, this demonstrated that specific IgG from the serum infusion had remained in circulation for more than a week. This is consistent with a half-life of murine IgG of approximately 8 days (32). We did not detect significant antigen-specific IgM in any of immunized animals at this time-point.

To understand if the development of subunit-specific GC B cells had been influenced by the serum injection, we determined the absolute number of splenic gp120- and gp41-specific GC B cells that had been induced in respective groups of mice. Here, we found that a distinct inhibition of gp120-specific GC B cells had occurred in both of the serum-infused groups (Figure 4D). This verified that the infused Env-specific and gp120-specific serum had similar capacity to suppress gp120-specific GC B cell responses. By contrast, inhibition of gp41-specific GC B cells had only occurred in the groups of mice that had received Env-specific serum. This suggested that gp41-specific GC B cell responses had been negatively regulated in the presence of high-affinity Env-specific antibodies that target the gp41 subunit, but not by antibodies that targeted the gp120 subunit.

Collectively, these data suggest that high-affinity antibodies at the level of a normal immune response can provide a negative feedback to repress the development of specific GC B cell responses, but that this only occurs if the antibodies and the GC B cells target the same or overlapping epitopes on Env.

## Discussion

Here, we performed a characterization of GC B cell responses to Env after a single immunization in mice, and subsequently addressed if antibodies have potential to regulate the development of GC B cells through an epitope-specific feedback mechanism. Our data suggest that single GCs are seeded by a polyclonal B cell population within a week after immunization with Env. To note, only two mice of six had developed distinct GCs at this time point. While we could not definitively rule out contamination from naïve B cells at this early time point, prior to clonal outgrowth, our data are consistent with the diverse early GC response after immunization of mice with chicken gammaglobulin, as has previously been shown (11). During the second week after immunization, varying degrees of clonal dominance is established in single GCs (Figure 2C). This coincides with peak frequency of total GC B cells in spleens of injected animals, and the detection of Env-specific GC B cells. To minimize the influence of non-cognate B cells that transport antigen to follicular dendritic cells or residual background from follicular B cells that did not participate in the GC reaction (33–35), we also made a qualitative approximation of clones in single GCs (Figures 2B,D). In this setting, we found that 9 of 14 single GCs contained between 1 and 4 distinct Vh1 fragments, where 3 GCs had potential to be fully monoclonal within the Vh1-family VDJ fragment length. During the third and fourth week after immunization, clonal dominance in single GCs had returned to levels that were indistinguishable from day 6. It was previously shown that tens to hundreds of individual B cell clones participate in the initial GC reaction (11). By the spectratyping approach used here, it was not possible to directly enumerate individual B cell clones but it was sufficient to approximate the relative clonality of single GCs at separate time points after injection of mice with Env.

After a single injection of mice with Env, we could demonstrate that up to 50% of the GC B cell response was focused on the gp41 subunit of Env. Consistent with these findings, gp41-specific plasma cells represent up of 50% of all Env-specific B cells after a booster injection (20). This suggests that GC B cells that develop in mice after a single injection of Env may differentiate into plasma cells after a subsequent booster injection. In line with this, the absence of V3-specific GC B cell development after a single injection with Env could therefore explain the absence of V3-specific plasma cells after a booster injection, as previously shown (13, 20).

Importantly, we proceeded to generate evidence that antibodies can feedback regulate the development of epitope-specific B cells. By injection of high-affinity polyclonal serum in mice at a time point where the endogenous GC response had been initiated, but prior to detection of Env-specific GC B cells, we found that preexisting antibodies to the gp120 subunit could repress gp120-specific but not gp41-specific GC B cells (Figure 4D). By contrast, infusion of Env-specific serum could repress both gp120 and gp41-specific GC B cell responses. Since V3-specific GC B cells had not developed after a single injection of mice with Env, we could not assess if also V3-specific GC B cell responses could be suppressed by a similar mechanism. Interestingly, infusion of Env in complex with a V3-specific Fab was recently shown to specifically suppress endogenous V3-directed antibody responses in Guinea pigs (36). This suggests that the development of V3-specific GC B cells may also be regulated by a similar antibody feedback-mediated mechanism as we here describe for gp41-specific GC B cells.

Since T cells are rapidly primed within the first days after antigenic challenge (37, 38), it is unlikely that priming of Tfh cells was affected by the day 4 serum injection. Moreover, presentation of antigenic peptides on MHC class II cannot directly explain a regulatory feedback mechanism that is dependent on the binding specificity of GC B cells. Similarly, a regulatory feedback mechanism that is dependent on the binding specificity of soluble antibodies is difficult to explain by engagement of the constant Fc-region of the infused IgG to the inhibitory Fc-gamma receptor IIb (39). In fact, a recent study demonstrated that antibody feedback of epitope-specific GCs during experimental antigen challenge act independently of Fc-gamma receptor engagement (40).

We therefore propose Env-specific B cell responses to HIV-1 Env are feedback regulated by epitope masking of antigen by high-affinity antibodies, and that this leads to a subsequent inability of low-affinity B cell clones with similar specificity to acquire stimulation *via* their BCR. In GCs, the antibody-mediated occlusion may occur on antigen that has been deposited on the FDC network, as was previously proposed by infusion of IgM (17). In our study, we investigated how early low-affinity GC B cells were affected by infusion of high-affinity IgG.

Clearly, additional research is required to fully understand how the epitope-specific GC B cell response is regulated during the gradual affinity increase and subsequent termination or differentiation of GC B cells during an endogenous immune response after vaccination with HIV-1 Env, but also if and how an antibody-based feedback can regulate the fate of memory B cells after re-challenge, as recently discussed (40–43).

Collectively, we provide data that strongly suggest that the development of GC B cells to a biologically relevant antigen is directly regulated by the presence of physiological levels of circulating antibodies. An affinity-dependent and antibody-mediated feedback to regulate affinity maturation of GC B cells has been suggested (17). We propose that this feedback acts on GC B cells only if they share the same or overlapping specificity as the circulating antibodies. Undoubtedly, the future development of well-defined mouse-derived monoclonal will allow for a more detailed investigation with regards to the biochemical and molecular properties of the inhibitory function of antibodies that target overlapping, partially overlapping, and non-overlapping epitopes of Env. Such data would be invaluable for the future designs of novel antigens for vaccination against HIV-1.

Importantly, the data presented here suggest that non-neutralizing or strain-specific neutralizing determinants on vaccine antigens have potential to suppress the development of bNab only if they share an overlapping binding site with these on Env. Our study therefore validates previous and on-going efforts to develop Env-based vaccine antigens with reduced exposure of non-neutralizing epitopes to the immune system (44–46), and we propose that it is crucial to focus these efforts on areas of Env where non-neutralizing epitopes overlap with broadly neutralizing epitopes.

## Ethics Statement

All animal experiments were pre-approved and performed in accordance with the Swedish Animal Welfare Act under protocols Dnr 234/12-dnr 11/13 (approved by Stockholms Norra djurförsöksetiska nämnd, Sweden) and Dnr A 59-15 (approved by Umeå försöksdjursetiska nämnd, Sweden).

## Author Contributions

MF: experimental design, performed experiments, analyzed data, and wrote the manuscript. MK: experimental design, analyzed data, and wrote the manuscript. LK, SS, JA: performed experiments.

## Conflict of Interest Statement

The authors declare that the research was conducted in the absence of any commercial or financial relationships that could be construed as a potential conflict of interest. The reviewer OB and handling Editor declared their shared affiliation and the handling Editor states that the process nevertheless met the standards of a fair and objective review.
